# Hula as a physical activity and social support intervention for sustained activity in female breast and gynecologic cancer survivors

**DOI:** 10.3389/fpsyg.2023.1190532

**Published:** 2023-10-24

**Authors:** Erin O. Bantum, Paulette M. Yamada, TeMoana Makolo, Herbert Yu, Ian Pagano, Natalie Subia, Catherine Walsh, Lenora W. M. Loo

**Affiliations:** ^1^Cancer Prevention in the Pacific, Population Sciences in the Pacific, University of Hawaii Cancer Center, University of Hawaii at Manoa, Honolulu, HI, United States; ^2^Kinesiology and Rehabilitation Science, College of Education, University of Hawaii at Manoa, Honolulu, HI, United States; ^3^Cancer Epidemiology, Population Sciences in the Pacific, University of Hawaii Cancer Center, University of Hawaii at Manoa, Honolulu, HI, United States; ^4^Cancer Biology Program, University of Hawaii Cancer Center, University of Hawaii at Manoa, Honolulu, HI, United States

**Keywords:** physical activity, cancer, culturally grounded interventions, cancer survivorship, social support

## Abstract

**Background:**

Physical activity improves health and psychosocial functioning for people who have been diagnosed with cancer. Native Hawaiians face disparities for some cancers, including breast cancer. Delivering culturally grounded interventions has the potential to improve enjoyment and adherence to the intervention. We sought to test the adherence and impact of a 6 month randomized wait-list controlled trial of hula.

**Methods:**

In this randomized wait-list controlled design people who had been diagnosed with breast or gynecologic cancers were invited to participate with other cancer survivors in a group based setting. Participants were randomized to begin hula immediately or after six months. Attendance was collected and heart-rate measured three times per session. In addition, demographic data, self-report psychosocial data, and biological data (findings will be reported elsewhere) were collected at three time points: baseline, 6 months, and 12 months. The study included six months of hula, twice per week, 60 min each session. In addition, participants committed to practice 60 min per week at home.

**Results:**

Participants in the study (*n* = 42) attended, on average, 72% of the sessions. Significant increase in moderate physical activity (*d* = 0.50, *p* = 0.03) was observed in the intervention versus control group. For the measures of intra-individual changes pre-and post-intervention, an increase in total physical activity were seen in the intervention group (*d* = 0.69, *p* = 0.003), daily caloric intake decreased (*d* = −0.62, *p* = 0.007), and a reduction in waist circumference (*d* = −0.89, *p* = 0.0002) that was sustained six months after completion of the intervention. Psychosocially, cognitive functioning significantly declined from baseline to 12 months (*d* = −0.50, *p* = 0.03), with role functioning improving (*d* = 0.55, *p* = 0.02), social constraints increasing (*d* = 0.49, *p* = 0.03), and financial difficulties improving (*d* = −0.55, *p* = 0.02).

**Conclusion:**

Sustainable physical activity is crucial to improve both the survival and quality of life of cancer survivors. Culturally grounded interventions, such as hula have the potential to increase the maintenance of physical activity. In addition, they create a support group where the benefits of people who have all experienced cancer can gather and garner those benefits of social support, too. This study was registered as a clinical trial through the National Cancer Institute (NCT02351479).

**Clinical trial registration:**

Clinicaltrails.gov, NCT02351479.

## Introduction

Cancer survivorship rates continue to rise in the US and are attributed to the aging population and advancing medical technologies (Miller et al., 2022). It is well-documented that physical activity is an essential aspect of health in cancer survivors as exercise attenuates the effects of cancer treatment and inactivity (Campbell et al., 2019). Epidemiologic studies have shown a strong association between physical activity and reduced mortality risk and from cancer (Schmid and Leitzmann, 2014). In fact, a meta-analysis of 71 cohort studies found a 13% reduction in mortality for cancer survivors who engaged in 2.5 h of moderate intensity physical activity every week, while those who exercised with greater volume (higher intensity or longer duration) had a 27% lower cancer mortality risk (Li et al., 2016). The reduced risk of mortality has been observed for many cancer types, including colorectum, prostate, endometrium, and breast (Stout et al., 2017).

In the current paper, we focus on the effects of physical activity within a social supportive context on female breast and gynecologic cancers survivors. Among breast cancer survivors, research has consistently shown that exercise reduces mortality of breast cancer (Holmes et al., 2005; Irwin et al., 2011; Friedenreich et al., 2020). This could be explained by the observation that cancer treatment and physical inactivity increases co-morbidity risk (Dieli-Conwright et al., 2016), while exercise attenuates/counteracts these effects (Dieli-Conwright et al., 2018). An observational study showed that patients diagnosed with breast cancer were free of metabolic syndrome before chemotherapy. Still, after 12 weeks of chemotherapy, 73% (of 86 patients) were newly diagnosed with metabolic syndrome as defined by lipidemia, hyperglycemia, and elevated proinflammatory cytokines, all of which are known to promote tumor progression (Dieli-Conwright et al., 2016). A follow-up randomized control trial showed that resistance and aerobic exercise reduced symptoms of metabolic syndrome and associated biomarkers in breast cancer survivors who completed primary cancer treatment (Dieli-Conwright et al., 2018). Of significance, central adiposity has been identified as more relevant in determining mortality risk than overall obesity (Zimta et al., 2019). In a large review of multiple meta-analyses found that, even when accounting for overall obesity, central obesity (such as hip-waist circumference, hip or waist, etc.) was associated with a significant increase in all-cause mortality (Jayedi et al., 2020). Some work in the area of dance for breast cancer patients has found improvements in hip circumference as a result of a 16 week intervention (Karkou et al., 2021). Also of significance, our prior work the area of dance for breast cancer patients has found improvements in waist circumference as a result of a 6 month hula intervention (Loo et al., 2019). Measures of central adiposity such as waist circumference have been shown to more strongly associate with all-cause mortality among breast cancer survivors than measures of overall obesity (George et al., 2014).

In addition to these positive biological benefits, psychosocial changes also occur with exercise. For example, exercise alters serotonin and dopamine levels (Lin and Kuo, 2013), which could affect mood or vice versa. Specifically, reduced serotonin levels after (moderate intensity) exercise are associated with a decreased stress sensitivity and a brighter outlook (Kiser et al., 2012). Breast cancer survivors who engage in exercise on their own have seen improvements in social functioning (Irwin et al., 2008; Cadmus et al., 2009; Craft et al., 2012; Mishra et al., 2012). In addition, serotonin and dopamine modulate perceived exercise exertion. Thus, consistent physical activity could enhance motivation and improve exercise tolerance (Marcora, 2008), ultimately improving adherence.

Moreover, exercising in a group setting fosters cohesion (Leach et al., 2019) which could provide even more benefit through social support. Social support has been found to play a vital role in the adjustment for cancer survivors. One of the seminal meta-analyses first published on the relationship between social support on mortality and health was conducted by Cassel in 1976 (Cassel, 1976). Soon after that publication, a longitudinal study examined the Alameda County Study Data. A link between social ties and mortality was found, independent of initial health status and health practices, including smoking, alcohol consumption, obesity, and physical activity (Berkman and Syme, 1979). Of significance, the magnitude of effect between social support and mortality was similar to that of smoking and obesity with mortality (Berkman and Syme, 1979; Vila, 2021).

While empirical evidence shows the importance of exercise, the adoption of physical activity among cancer survivors is challenging (Avancini et al., 2020). Although the positive impact of physical activity has been strongly demonstrated, in 2020, 35.5% of cancer survivors aged 18 years and older reported no physical activity in their leisure time (National Cancer Institute, 2022). Promoting physical activity rooted in a group’s culture and tradition may effectively induce sustainable physical activity (Kaholokula et al., 2021). For example, Boing et al. (2020) studied belly dancing as a mode of physical activity in a study that focused on breast cancer survivors. Belly dance connects mind and body through movement and incorporates expressive movements that preserve femininity, security, and the sound of traditional Arabic music (Boing et al., 2020). Cultural identity encompasses ethnic pride and knowledge, involvement in ethnic practices, and a cultural commitment or feeling of belonging to one’s ethnic group (Park and Huang, 2010). Cultural identity has been demonstrated as being relevant to mental and physical heath (Mossakowski, 2018). Engaging in health promotion activities that are culturally grounded and derived brings together not simply the relevance of physical activity, may also bring a sense of pride in one’s culture. In looking at differences in exercise by ethnic group, there is very little work in this area. Some work comparing European-American and Latin-American cancer survivors, has demonstrated that European-Americans were more likely to be engaging in exercise after treatment (Ashing-Giwa et al., 2010), although more work in this area is certainly needed.

In the Native Hawaiian culture, hula, or dance, encompasses their culture, identity, history and existence. Through hula, Native Hawaiians tell stories of religion (to honor gods/goddesses, offer prayer) and culture (Ka ‘Imi Na auao O Hawaii Nei Institute, 2019, accessed 2023). Hula is a source of pride, as it is used to educate their children and is used to pass down their history, stories and wisdom to the next generation (Ng, 2022). After western contact, in approximately 1778, there were efforts to forbide hula, to include a ban of public hula in 1830 and many other attempts to discourage hula, it has always continued (Ka ‘Imi Na auao O Hawaii Nei Institute, 2019, accessed 2023). Since hula is deeply rooted (or engrained) in Native Hawaiian culture, we implemented hula as the form of physical activity in this study. In a previous pilot study, we demonstrated feasibility and adherence for a 6-month hula ‘auana intervention (a modern, free-flowing dance performed with Hawaiian music). This study maintained an average attendance of 84% during the 6-month intervention. Moreover, the study showed sustainability, as a subset of participants continued to participate in the program for two years post-intervention under the direction of the same teacher (Loo et al., 2019). Here we used the pilot study design to evaluate the effects of a 6-month hula ‘auana intervention on both psychological and physical health in a waist-list control trial. We hypothesized that people randomized into the treatment group would experience better mood, improvements in fatigue, and fewer social constraints. In addition, we hypothesized a reduction in central adiposity (measured via hip-waist circumference) compared to participants randomized into the wait-list control group.

## Methods

### Study participants

**Figure 1:**
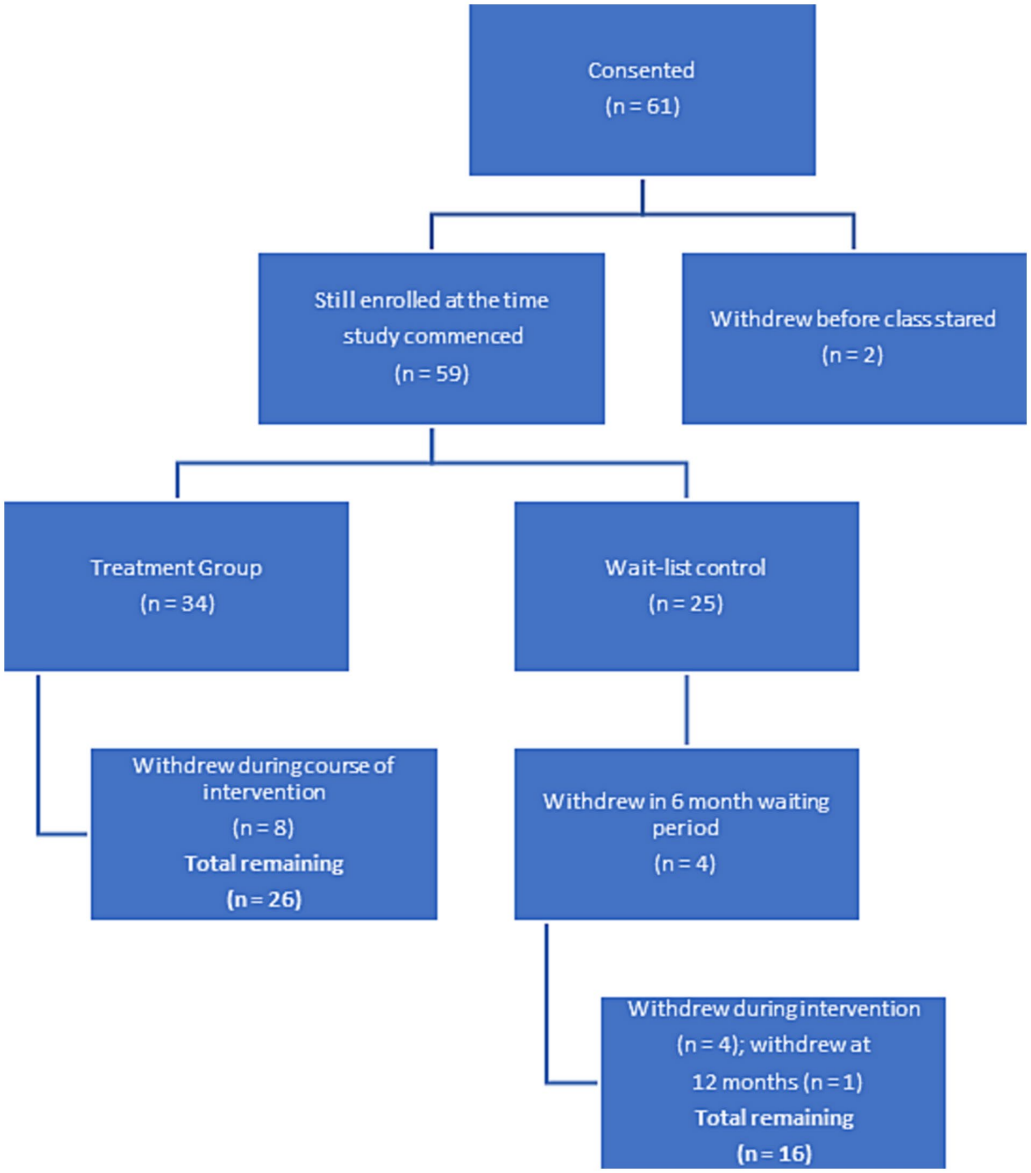
Flow chart for study participant adherence.

In this randomized wait-list controlled study, female breast and gynecologic cancer survivors who had completed primary treatment at least two months prior were recruited for this study (*n* = 61). Although this was designed to be a fully powered randomized controlled trial, due to difficulty recruiting, we conducted a pilot study. Participants were required to have clearance from their physician to engage in the study. Potential participants were identified through oncology treatment clinics and hospitals on O‘ahu and the University of Hawai‘i Cancer Center website, in addition to public events, such as senior fares. Potential participants spoke to a project coordinator over the phone to determine if the eligibility criteria were met. If the study criteria were met, the potential participant was mailed a packet containing questionnaires on demographic and validated self-report measures, a stool kit, and an informed consent. Randomization occurred *via* a computer program after the informed consent was complete. By the time the wait-list group was ready to begin the intervention (6 months after the treatment group) a number of people had either moved or were no longer interested in person, which resulted in an unequal number of participants in each group. An in-person assessment appointment was also scheduled to address any questions regarding the questionnaires and consent form and ensure the study documents’ completion. During this appointment, anthropometric, fecal, saliva, and blood samples were collected. Participants were also required to complete a 24-h recall dietary assessment (Vereecken et al., 2008). Details of each instrument are presented below. Data (aside from demographics) and biospecimens, were collected at three time points (baseline, six months, and 12 months). Eligible participants were randomized to a study arm (e.g., Intervention or Wait-list Control). The Western Institutional Review Board approved the study. Figure 1 demonstrates attrition throughout the study time period.

### Study design

For the intervention group, participants were required to attend an in-person hula dance class at the chosen location, the University of Hawaii Cancer Center or Pali Momi Medical Center, twice a week for 60 min each session. In addition, participants agreed to practice at home for a minimum of 15 min, 3 times a week for six months (Loo et al., 2019). The goal was to include the recommendation by the Centers for Disease Control (2023) (accessed 2023) of incorporating 150 min of moderate to vigorous physical activity each week. Each 60-min Hula dance class included a warm-up (10 min), conditioning (40 min), and cool-down (10 min) period. Heart rate was measured using a pulse oximeter three times during each class (start of class, mid-conditioning, end of class). The target heart rate of 50–70% of the maximum heart rate (HRmax = 220-age) during the mid-conditioning period of the class was intended to achieve the moderate-intensity physical activity during class. Participant attendance was recorded to track adherence and participation. If a participant in the intervention group missed two consecutive classes, they were contacted by the project coordinator to encourage attendance. This study was designed as a fully powered randomized-controlled trial, although given difficulty in recruitment, the sample size was smaller than anticipated.

### Data collected

Data were collected at three time points: baseline (T1), month-6 (T2), and month-12 (T3). Participants were mailed questionnaire packets and then arrived at the clinic for physical assessments. At that time, the project coordinator reviewed the data and, any missing data was sought, if it was data the participants had not intentionally left blank. For participants randomized into the treatment condition they were assessed just before the beginning of the intervention, immediately after (T2), and six months after the completion of the intervention (T3). For wait-list control participants, they were assessed six months before their intervention began (T1), just before the intervention began (T2) and after the completion of the intervention (T3). The following information was collected:

### Anthropometric measures

Height, weight, body mass index (BMI), waist circumference, hip circumference, and waist/hip circumference ratio (WHR) were measured using a tape measure by trained personnel responsible for taking these measurements at each assessment time point.

### Questionnaires

#### Demographic and health information

Participants completed a questionnaire providing demographic (e.g., age, race/ethnicity, marital status, education) and health information (e.g., cancer site, cancer stage at diagnosis; see Table 1).

**Table 1:** Demographic differences between groups.

Variable	Value	Total		Hula		Control		*p*
		*N* = 42		*N* = 26		*N* = 16		
		*n*	Total %	*N*	Total %	*n*	Total %	
Age	40–60	13	31.0	10	38.5	3	18.8	0.42
	61–70	20	47.6	11	42.3	9	56.3	
	71–85	9	21.4	5	19.2	4	25.0	
Race	Caucasian	8	19.0	5	19.2	3	18.8	0.94
	Chinese	5	11.9	3	11.5	2	12.5	
	Japanese	11	26.2	8	30.8	3	18.8	
	Native Hawaiian	12	28.6	7	26.9	5	31.3	
	Other	6	14.3	3	11.5	3	18.8	
Marital status	No Partner	15	35.7	9	34.6	6	37.5	0.99
	Partner	27	64.3	17	65.4	10	62.5	
Education	No Bachelor’s Degree	14	33.3	8	30.8	6	37.5	0.62
	Bachelor’s Degree	16	38.1	9	34.6	7	43.8	
	Higher Degree	12	28.6	9	34.6	3	18.8	
Cancer site	Breast	35	83.3	21	80.8	14	87.5	0.69
	Other	7	16.7	5	19.2	2	12.5	
Cancer stage	0–1	24	57.1	16	61.5	8	50.0	0.53
	2–3	18	42.9	10	38.5	8	50.0	
Study facility	Pali Momi Hospital	20	47.6	13	50.0	7	43.8	0.76
	UH Cancer Center	22	52.4	13	50.0	9	56.3	

#### Exercise

The Women’s Health Initiative Physical Activity Questionnaire (Meyer et al., 2009), a 30-item instrument originating from the Women’s Health Initiative Study (WHI) was used to measure exercise type, duration, frequency, and intensity during the intervention and after the intervention was complete. This would include any physical activity from hula or other activities.

#### Diet

Three days of 24-h diet recall (Vereecken et al., 2008) were collected and used to calculate total caloric intake to be compared to total MET expenditure. The version used was online, although if participants had difficulty completing it online, or did not have access to the internet, the project coordinator would call the participant and collect the data over the phone, which she directly entered into the online survey. The 24-h recall data was analyzed using the Healthy Eating Index 2015 (Guenther et al., 2013) to assess dietary behavior change over time to determine if participants experienced multiple health behavior changes during the study. The online version has strong agreement with the standard 24-h recall (Timon et al., 2016).

#### Health-related quality of life

The European Organization for Research and Treatment of Cancer QLQ-C30 is a 30-item measure assessing patients’ quality of life (Aaronson et al., 1993). The measure includes three scales: global health status, functional status (physical, role, emotional, cognitive, and social), and physical symptoms. The index has demonstrated face and convergent validity, internal consistency, and test–retest reliability (Hjermstad et al., 2016).

#### Fatigue

The Brief Fatigue Inventory (BFI) is a 10-item measure assessing both the severity of fatigue and the impact of fatigue on daily functioning during the last 24-h period (Mendoza et al., 1999). The BFI has been well validated with strong internal consistency (Mendoza et al., 1999).

#### Depression

The Centers for Epidemiological Studies Depression Scale (CES-D) consists of 20 items yielding a single score used to indicate the presence of depression (Radloff, 1977). This measure is well used in studies with people who have been diagnosed with cancer and has strong internal consistency and test–retest reliability (Miller et al., 2013).

#### Social constraints

The 10-item Social Constraints Scale has been used to measure how people adjust and cope with a cancer diagnosis. Social constraints are associated with avoidance of talking and thinking about cancer, intrusive thoughts and uncertainty about cancer, poor marital quality, and heightened psychological distress (Lepore and Ituarte, 1999; Norton et al., 2011). Internal consistency for this measure has been found to be high (Davis et al., 2019).

#### Cognitive functioning

The Functional Assessment of Cancer Therapy-Cognitive Subscale (FACT-cog), is a 42-item measure consisting of two subscales: cognitive deficiency and cognitive capability (Lai et al., 2009). Internal consistency and test–retest reliability has been found for this scale (Cheung et al., 2013).

#### Mood

Profile of Mood States (POMS). It includes 65 adjectives describing feelings grouped in six mood dimensions: tension-anxiety, depression-dejection, anger-hostility, vigor-activity, fatigue-inertia, and confusion-bewilderment. The POMS has demonstrated good reliability and validity (Curran et al., 1995).

### Statistical analyses

Data was entered into an electronic database by the project coordinator and spot checked by the first author (EOB). To test for intra-individual differences pre-and post-intervention for each assessment, we ran mixed (both between-and within-subjects variables) linear regression models (Singer and Willett, 2003). The outcomes were dietary, exercise, and psychological assessments; and we ran a separate model for each. The predictors in all models were randomization group (hula intervention vs. wait-list control), time of assessment (at baseline [T1] and at six months post-hula intervention [T2]), and the group by time interaction. The interaction term tested the hula intervention effect. Age, cancer site, and time since diagnosis were included as covariates. The randomization group and the covariates were time-invariant (between-subjects) effects, whereas time of assessment and the group by time interaction were time-varying (within-subjects) effects.

**Figure 2:**
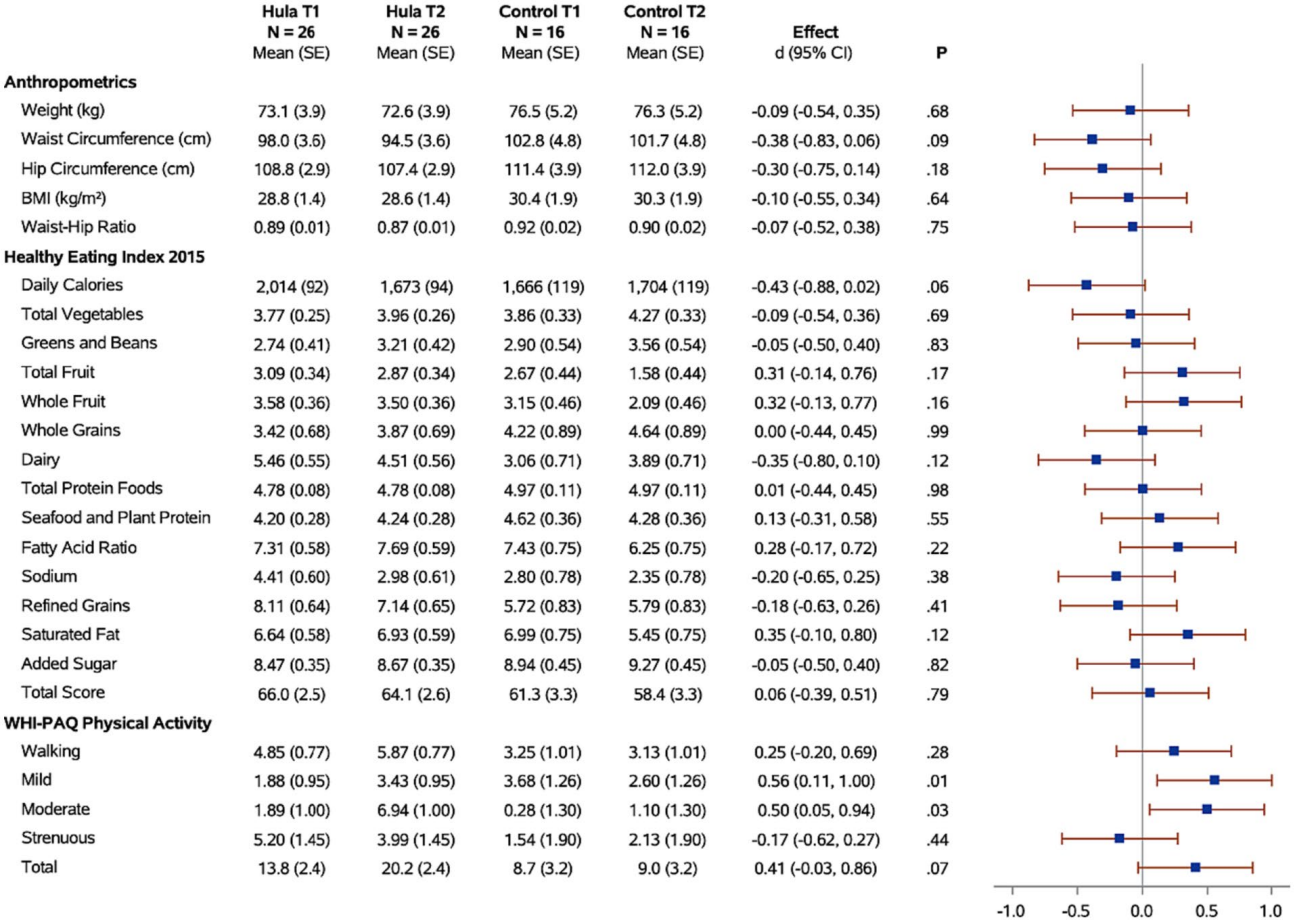
Forest plots of the standardized hula intervention effect (Cohen’s d) for the diet and exercise measures with 95% confidence intervals are shown. Results are adjusted for age, cancer site, and time since diagnosis. Sample sizes (*N*), means, and standard errors (SE) are shown for the hula intervention group and the control group at baseline (T1) and six months (T2).

**Figure 3:**
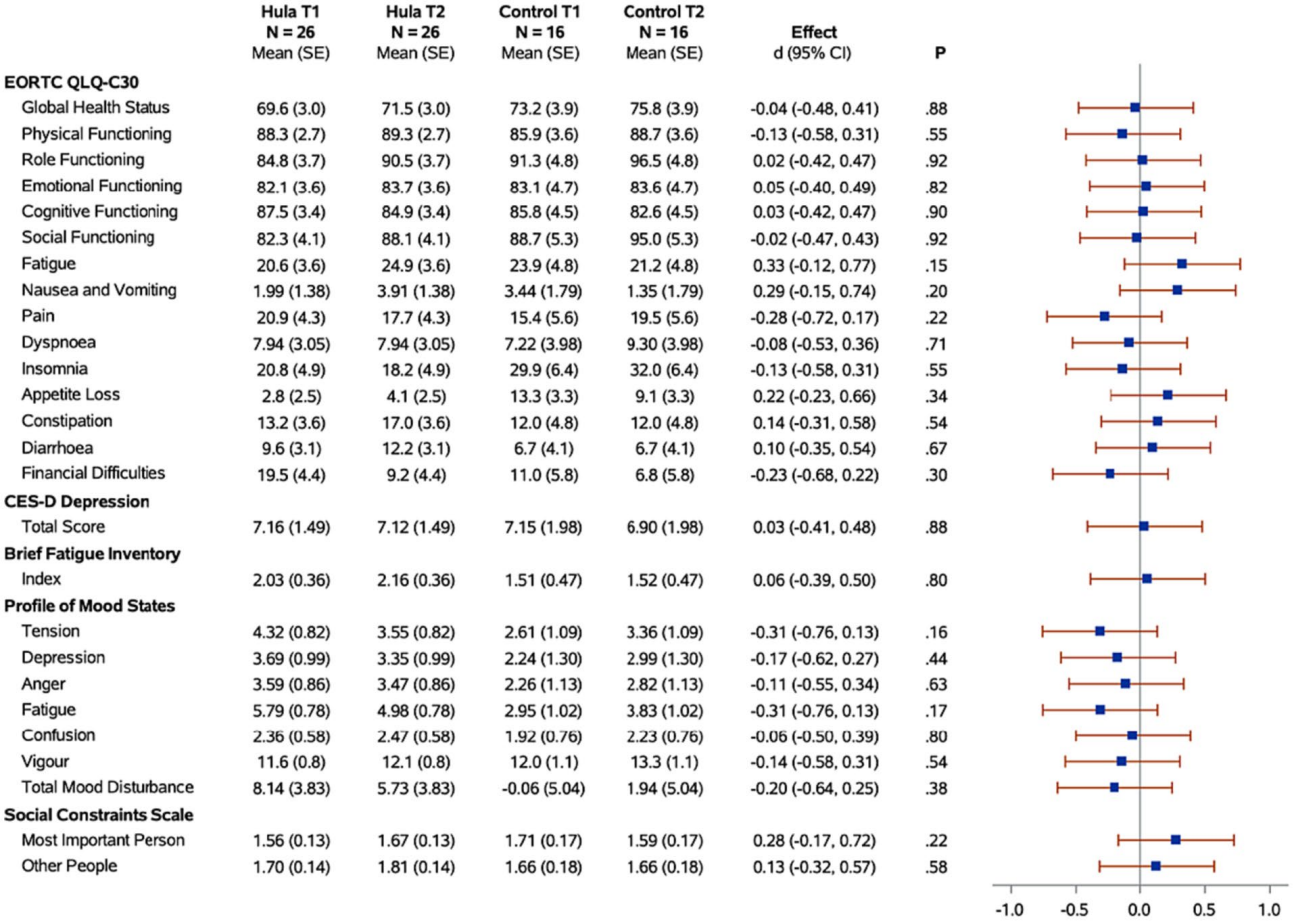
Forest plots of the standardized hula intervention effect (Cohen’s d) for the psychological measures with 95% confidence intervals are shown. Results are adjusted for age, cancer site, and time since diagnosis. Sample sizes (*N*), means, and standard errors (SE) are shown for the hula intervention group and the control group at baseline (T1) and six months (T2).

Descriptive statistics for participants who entered and completed the study are shown in Table 1. Table 1 shows only the women who completed the study, comparing those randomized to the hula intervention group and those randomized to the wait-list control group. Forest plots for the hula intervention effect in those who completed the study (*N* = 42) are shown in Figures 2, 3. Figure 2 has the dietary and exercise assessments, and Figure 3 has the psychological assessments. The *p*-values for comparing the groups are from Fisher’s exact test. The *p*-values for assessing the hula intervention effect are from the interaction term in the mixed regression model. The SAS 9.4 software (SAS Institute Inc., Cary, NC) performed for all analyses. Given the small sample size, the study was underpowered. Because of this, all analyses were exploratory, with results being assessed as trends.

## Results

In this study, we recruited 61 individuals into the intervention (Figure 1; Table 1). The mean age of participants was 65 years, with an age range of 40–84 years. Among the 61 participants at baseline, the self-reported racial/ethnic distribution of study participants was 22% Hawaiian, 27% Caucasian, 30% Japanese or Okinawan, 27% Chinese or Taiwanese, and 12% other (including Filipino). Sixty-five percent of the participants indicated they were married and the majority (67%) of participants had a college education (Bachelor’s Degree or higher). The distribution of the participants by cancer type was as follows, 83% of the participants had breast cancer, and 17% had other cancer (ovarian, cervical, and endometrial).

Among the 61 individuals who started the study, 42 (68.8%) completed the 12-month study. Participants attended an average of 72% of the classes during the course of the six-month intervention. Our preliminary study findings demonstrated that 92% of the Native Hawaiian participants completed the study, in contrast to 50% of Caucasian and 61% of Japanese participants completing the study (Supplementary Table S1).

**Figure 4:**
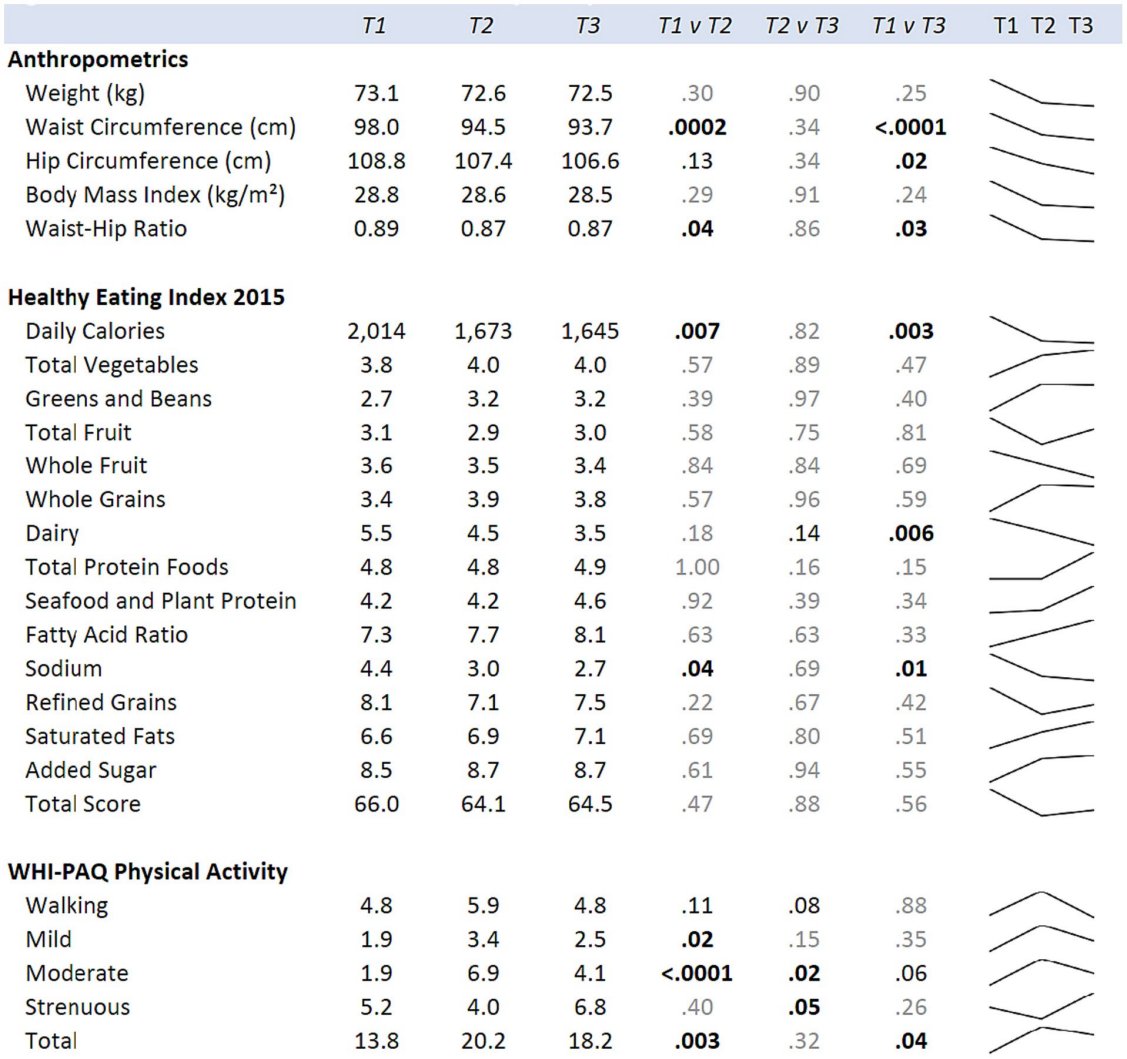
Means for the hula intervention group at baseline (T1), six months (T2), and 12 months (T3) are shown. *p*-values comparing each time point are from mixed regression models with the diet and exercise measures as the outcomes, and time as the predictor. Results are adjusted for age, cancer site, and time since diagnosis. Sparklines show the longitudinal trend.

Changes in physical activity were measured using the WHI questionnaire. When examining the effects of the hula intervention on the treatment group versus the wait-list control group (Figure 2), we observed a non-significant increase in total physical activity (*d* = 0.41, 95% CI = −0.03, 0.86; *p* = 0.07) that was observed in the treatment group versus the wait-list control group. However, when evaluating by physical activity categories, e.g., mild, moderate, and strenuous physical activity, there was a significant increase in mild (*d* = 0.56, 95% CI = 0.11, 1.00; *p* = 0.01) and moderate (*d* = 0.50, 95% CI = 0.05, 0.94; *p* < 0.03) physical activity in the treatment group compared to the wait-list control group. There was no significant difference in the strenuous physical activity levels between the two groups. When examining the intra-individual change (T1 vs. T2) for the intervention group (*n* = 26) pre-versus post-intervention, a significant increase was observed for total (*p* = 0.003), mild (*p* = 0.02), and moderate (*p* < 0.0001) physical activity (Figure 4). The levels of physical activity decreased following the end of the intervention (month-12), however, it did remain higher than the baseline levels for the total physical activity (T1 vs. T3; *p* = 0.04) (Figure 4). When examining intra-individual change for the entire sample of participants (*n* = 42) that completed the hula program, there was a highly significant outcome on total physical activity (*p* < 0.0001), moderate exercise (*p* < 0 0.0001), and mild exercise (*p* < 0.0001). There was no significant change in strenuous physical activity (*p* = 0.59) (Supplementary Figure S1).

When examining for significant changes pre-and post-treatment (T1 and T2) in the anthropomorphic measures between the hula intervention treatment versus the wait-list control groups, we did not observe a significant difference in the changes between the two groups (Figure 2). Although not significant, we observed a trend toward a reduction in all anthropomorphic measures in the baseline (T1) versus 6-month (T2) comparison between treatment and wait-list control participants. When examining the intra-individual change (T1 vs. T2) for the intervention group (*n* = 26) pre-versus post-intervention, a significant reduction in the waist circumference (*p* = 0.0002) and waist hip ratio (*p* = 0.04) was observed (Figure 4). This reduction was sustained through month-12 (T3). When examining intra-individual change for the entire sample of participants (*n* = 42) that completed the hula program, there was a highly significant reduction in waist and hip circumference (*p* = 0.0009 and *p* = 0.009, respectively) (Supplementary Figure S1). There were no significant changes observed for weight, BMI, or WHR.

When examining for significant changes pre-and post-treatment (T1 and T2) in the dietary measures, using the HEI 2015, between the hula intervention treatment versus the wait-list control groups, we did not observe a significant difference in the changes between the two groups (Figure 2). When examining the intra-individual change (T1 vs. T2) for the intervention group (*n* = 26) pre-versus post-intervention, a significant reduction in the daily calories (*p* = 0.007) and sodium intake (*p* = 0.04) were observed (Figure 4). This reduction was sustained through month-12 (T3). When examining intra-individual change for the entire sample of participants (*n* = 42) that completed the hula program, there were no significant changes in dietary intake aside from an increase in saturated fat intake (*p* = 0.04) (Supplementary Figure S1).

**Figure 5:**
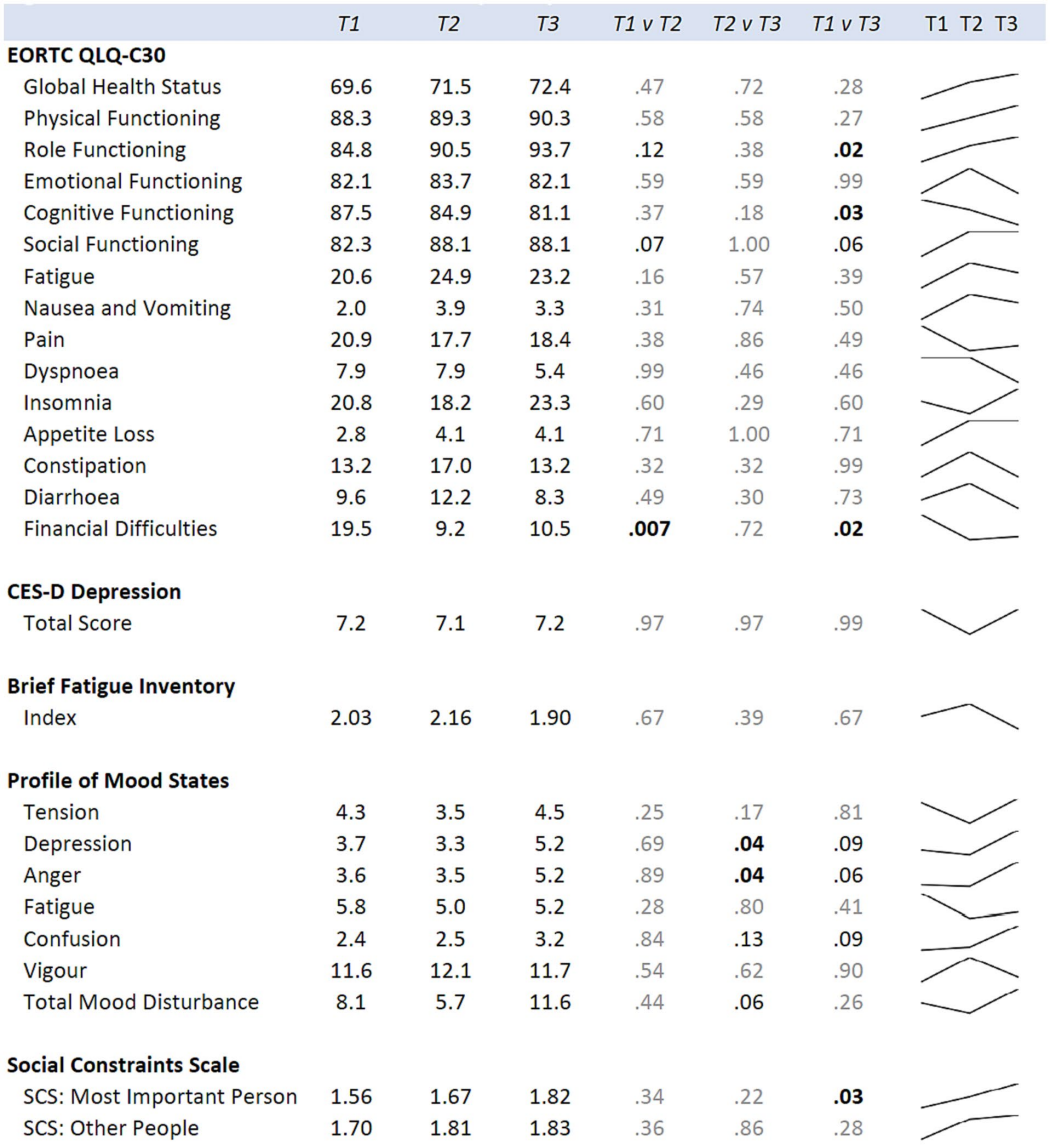
Means for the hula intervention group at baseline (T1), six months (T2), and 12 months (T3) are shown. *p*-values comparing each time point are from mixed regression models with the psychological measures as the outcomes, and time as the predictor. Results are adjusted for age, cancer site, and time since diagnosis. Sparklines show the longitudinal trend.

When examining for significant changes pre-and post-treatment (T1 and T2) in the psychosocial effects of the intervention, between the treatment versus wait-list control groups (Figure 3), we did not observe a significant difference in the changes between the two groups in any of the variables of interest: depression, fatigue, health-related quality of life, mood, or social constraints (Figure 3). However, in the intra-individual measures for the treatment group, of note at the month-12 time-point (T3), six months post intervention, participants kept making many gains (Figure 5). Psychosocially, there was a significant longitudinal improvement (over the three time-points) of role functioning over time (*p* = 0.02), although there was an increase in social constraints with the most important person in their lives (*p* = 0.03) as measured in the social constraints scale (Figure 5). There were observed significant decline in measures of cognitive functioning based on the EORTC-Q30 assessment from baseline to 12 months (*p* = 0.03). When examining intra-individual change for the entire sample of participants (*n* = 42) that completed the hula program, there were a no significant changes in the psychosocial measures (Supplementary Figure S2).

## Discussion

The aim of this study was to assess feasibility and psychosocial outcomes of a six month hula intervention for breast and gynecologic cancer survivors on the island of Oahu, which, like the state at large, includes ethnically diverse residents. We hypothesized that people randomized into the treatment group would experience better mood, improvements in fatigue, and fewer social constraints. In addition, we hypothesized a reduction in central adiposity (measured *via* waist circumference) compared to participants randomized into the wait-list control group. Observational and randomized controlled trials indicate that physical activity is effective in reducing the risk of developing primary breast cancer (Bianchini et al., 2002a,b), as well as recurrence and death following treatment for breast cancer (Holmes et al., 2005; Irwin et al., 2008, 2011; Ballard-Barbash et al., 2012). A positive impact on social functioning has been observed in exercise studies, and there is hope that with a fully powered study we could see a positive impact. In speaking with the participants in the study, this is one of the most important benefits they describe.

Although the statistical power was not achieved to study outcomes in these feasibility studies, the findings strongly support the importance of additional research on better understanding the role of culturally grounded physical activity in increasing interest and adherence to exercise regimens for cancer survivors. The initial findings point to early feasibility and adherence, although there are remaining questions around disseminating the intervention throughout the larger community as well as the particular impact of a culturally-relevant intervention for Native Hawaiian or Other Pacific Islander (NHOPI) women. Given that there are differences in incidence and mortality from multiple cancer types, the efficacy could be more impactful for this population as this intervention comes from the same cultural vantage point as the participants.

A large meta-analysis examining the effects of physical activity on cancer mortality found that pre-diagnostic and post-diagnostic physical activity was associated with reduced risk of breast cancer-specific mortality (HR = 0.86, 95%CI 0.78–0.94 and HR = 0.63, 95%CI 0.50–0.78, respectively) (Friedenreich et al., 2020). Using heart rate measurements in the current study, we showed that participants performed hula at a moderate intensity. Since we used the 220-age method to determine intensity, this may have underestimated the participants’ maximal heart rate thereby overestimating their actual physical exertion during the intervention. Notably even if hula intensity was lower than intended, 6 months of hula significantly reduced waist and hip circumference. Hula lines up with the minimum required intensity required to positively impact mortality risk (Lee, 2019). This is highly significant as individuals of Native Hawaiian descent have increased risk for many health conditions, including cardiovascular disease (Aluli et al., 2007), type 2 diabetes (Aluli et al., 2009), and an increased cancer mortality risk (Taparra et al., 2022). Furthermore, results of this study suggest that use of culturally relevant physical activity could help specific groups of patients initiate and maintain physical activity. Integration of different modes of exercise may be necessary to attract participants to physical activity programs, specifically supporting culturally grounded types of activities within ethnic minority communities. As we continue to support survivors through movement, it is important to consider all types of activity as a strong connection to the activity could drive motivation for long term participation.

There are many limitations of note in the current study. The study design included individuals who could attend a lengthy intervention at set times, multiple instances per week. This inherently excludes many people who could benefit from the intervention if they could not commit to the structured class times and frequency. The study took place on Oahu, which is just one of the many Hawaiian islands. Both sites on the island of Oahu were in the most populated area of the island and state (Honolulu). This certainly excluded many individuals who could benefit from the study. In addition, the study did not reach participant numbers to be powered to look at outcomes. More resources would be needed to do this, although given these initial findings, this is a next step in this work. Although the impact of physical activity on mortality is one of the primary reasons we conducted this work, given the length of the study, we did not look at survival as an outcome.

Adherence is truly the sticking point when it comes to all types of behavior change, including exercise. Engaging in what we enjoy is what leads to lasting change and lasting change is what leads to impact that is truly life changing. As a field we must do a better job at not only demonstrating that exercise and more informally, all forms of physical activity, is beneficial. It must be incorporated into our lives. Work such as this and that of our colleagues is crucial in regards to identifying interventions that will have lasting effects. Culturally grounded interventions, such as hula, are unique to certain populations, and indigenous exercise from all places geographically, has some similarities. Some of these important aspects could be music and movement that tells a story, a group component, a sense of reverence or appreciation for what is being practiced, and asymmetrical movement. Future work that continues to identify components that lead to lasting change is necessary.

## Data availability statement

The original contributions presented in the study are included in the article/Supplementary material, further inquiries can be directed to the corresponding author.

## Ethics statement

The study involving humans was approved by Western Internal Review Board (WIRB). The studies were conducted in accordance with the local legislation and institutional requirements. The participants provided their written informed consent to participate in this study.

## Author contributions

EB and LL contributed to the design, implementation, interpretation and write up of the results, in addition to the planning of the manuscript flow. PY contributed to the paper manuscript flow plan, writing up the manuscript, and editing other versions. TM contributed to the intervention design and to teaching the intervention. HY contributed to the study design and implementation and editing of the manuscript. IP contributed to the data analysis and interpretation and to writing of these sections in the manuscript. NS created tables and edited the manuscript. CW edited the manuscript. All authors contributed to the article and approved the submitted version.
