# Supplementary material for: Hula as a physical activity and social support intervention for sustained activity in female breast and gynecologic cancer survivors
Source: Front Psychol. 2023 Oct 24;14:1190532. doi: 10.3389/fpsyg.2023.1190532 (PMC10629222; doi:10.3389/fpsyg.2023.1190532)
Supplement: Supplementary file 1 [file Data_Sheet_1.pdf]

Supplemental Figure Legends

Supplemental Figure 1. Forest plots of the standardized hula intervention effect (Cohen's d) for the diet and exercise measures with 95% confidence intervals are shown. Results are adjusted for age, cancer site, and time since diagnosis. Sample sizes (N), means, and standard errors (SE) are shown for the hula intervention group at baseline (T1) and six months (T2), and for the wait list group at six months (T2) and 12 months (T3).

Supplemental Figure 2. Forest plots of the standardized hula intervention effect (Cohen's d) for the psychological measures with 95% confidence intervals are shown. Results are adjusted for age, cancer site, and time since diagnosis. Sample sizes (N), means, and standard errors (SE) are shown for the hula intervention group at baseline (T1) and six months (T2), and for the wait list group at six months (T2) and 12 months (T3).

Supplemental Table 1. Characteristics of participants that entered and completed the study

		TOTAL N = 61		COMPLETED N = 42		WITHDRAWN N = 19		
**Variable**	**Value**	**N**	**Col %**	**N**	**Col %**	**N**	**Col %**	**P-value***
Age	40-60	24	40.0	13	31.0	11	61.1	0.09
	61-70	26	43.3	20	47.6	6	33.3	
	71-85	10	16.7	9	21.4	1	5.6	
Race	Caucasian	16	26.7	8	19.0	8	44.4	0.09
	Chinese	6	10.0	5	11.9	1	5.6	
	Japanese	18	30.0	11	26.2	7	38.9	
	Native Hawaiian	13	21.7	12	28.6	1	5.6	
	Other	7	11.7	6	14.3	1	5.6	
Marital Status	No Partner	21	35.0	15	35.7	6	33.3	0.99
	Partner	39	65.0	27	64.3	12	66.7	
Education	No Bachelor's Degree	20	33.3	14	33.3	6	33.3	0.94
	Bachelor's Degree	22	36.7	16	38.1	6	33.3	
	Higher Degree	18	30.0	12	28.6	6	33.3	
Cancer Site	Breast	50	83.3	35	83.3	15	83.3	0.99
	Other	10	16.7	7	16.7	3	16.7	
Cancer Stage	0-1	34	56.7	24	57.1	10	55.6	0.99
	2-3	26	43.3	18	42.9	8	44.4	
Study Facility	Pali Momi Hospital	31	50.8	20	47.6	11	57.9	0.58
	UH Cancer Center	30	49.2	22	52.4	8	42.1	

* p-values are based on Fisher’s exact test

	Hula T1 N = 26 Mean (SE)	Hula T2 N = 26 Mean (SE)	Wait List T2 N = 16 Mean (SE)	Wait List T3 N = 16 Mean (SE)	Effect d (95% CI)	P
**Anthropometrics**						
Weight (kg)	73.1 (3.9)	72.6 (3.9)	76.3 (5.2)	75.5 (5.2)	-0.39 (-0.84, 0.05)	.08
Waist Circumference (cm)	98.0 (3.6)	94.5 (3.6)	101.7 (4.8)	100.3 (4.8)	-0.78 (-1.23, -0.33)	.0009
Hip Circumference (cm)	108.8 (2.9)	107.4 (2.9)	112.0 (3.9)	109.5 (3.9)	-0.60 (-1.05, -0.16)	.009
BMI (kg/m^2)	28.8 (1.4)	28.6 (1.4)	30.3 (1.9)	30.0 (1.9)	-0.42 (-0.86, 0.03)	.07
Waist-Hip Ratio	0.89 (0.01)	0.87 (0.01)	0.90 (0.02)	0.91 (0.02)	-0.15 (-0.60, 0.29)	.50
**Healthy Eating Index 2015**						
Daily Calories	2,014 (92)	1,673 (94)	1,704 (119)	1,909 (119)	-0.15 (-0.60, 0.29)	.50
Total Vegetables	3.77 (0.25)	3.96 (0.26)	4.27 (0.33)	3.40 (0.33)	-0.28 (-0.73, 0.16)	.21
Greens and Beans	2.74 (0.41)	3.21 (0.42)	3.56 (0.54)	3.30 (0.54)	0.05 (-0.39, 0.50)	.81
Total Fruit	3.09 (0.34)	2.87 (0.34)	1.58 (0.44)	2.30 (0.44)	0.18 (-0.27, 0.63)	.43
Whole Fruit	3.58 (0.36)	3.50 (0.36)	2.09 (0.46)	3.50 (0.46)	0.44 (-0.01, 0.89)	.06
Whole Grains	3.42 (0.68)	3.87 (0.69)	4.64 (0.89)	4.44 (0.89)	0.04 (-0.40, 0.49)	.84
Dairy	5.46 (0.55)	4.51 (0.56)	3.89 (0.71)	3.34 (0.71)	-0.30 (-0.74, 0.15)	.19
Total Protein Foods	4.78 (0.08)	4.78 (0.08)	4.97 (0.11)	4.97 (0.11)	0.01 (-0.44, 0.45)	.98
Seafood and Plant Protein	4.20 (0.28)	4.24 (0.28)	4.28 (0.36)	4.39 (0.36)	0.05 (-0.40, 0.50)	.82
Fatty Acid Ratio	7.31 (0.58)	7.69 (0.59)	6.25 (0.75)	7.80 (0.75)	0.34 (-0.11, 0.79)	.13
Sodium	4.41 (0.60)	2.98 (0.61)	2.35 (0.78)	1.73 (0.78)	-0.42 (-0.87, 0.03)	.07
Refined Grains	8.11 (0.64)	7.14 (0.65)	5.79 (0.83)	6.35 (0.83)	-0.07 (-0.52, 0.38)	.75
Saturated Fat	6.64 (0.58)	6.93 (0.59)	5.45 (0.75)	7.60 (0.75)	0.47 (0.02, 0.92)	.04
Added Sugar	8.47 (0.35)	8.67 (0.35)	9.27 (0.45)	8.97 (0.45)	-0.04 (-0.49, 0.41)	.86
Total Score	66.0 (2.5)	64.1 (2.6)	58.4 (3.3)	62.1 (3.3)	0.10 (-0.35, 0.55)	.65
**WHI-PAQ Physical Activity**						
Walking	4.85 (0.77)	5.87 (0.77)	3.13 (1.01)	3.75 (1.01)	0.35 (-0.09, 0.80)	.12
Mild	1.88 (0.95)	3.43 (0.95)	2.60 (1.26)	6.62 (1.26)	1.18 (0.74, 1.63)	<.0001
Moderate	1.89 (1.00)	6.94 (1.00)	1.10 (1.30)	3.94 (1.30)	0.93 (0.49, 1.38)	<.0001
Strenuous	5.20 (1.45)	3.99 (1.45)	2.13 (1.90)	2.09 (1.90)	-0.12 (-0.57, 0.32)	.59
Total	13.8 (2.4)	20.2 (2.4)	9.0 (3.2)	16.4 (3.2)	0.92 (0.47, 1.36)	<.0001

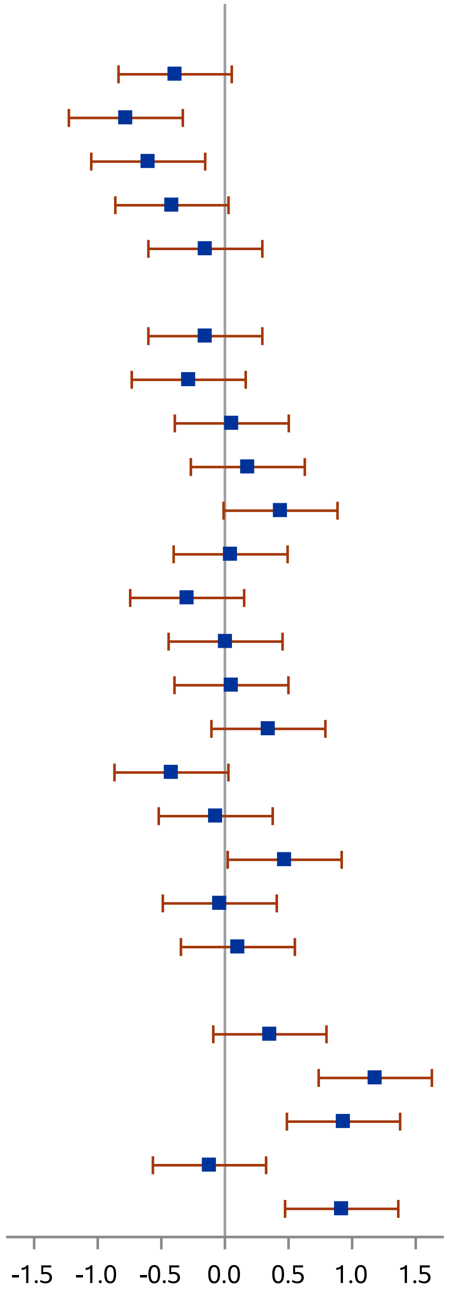

Supplemental Figure 1

	Hula T1<br>N = 26<br>Mean (SE)	Hula T2<br>N = 26<br>Mean (SE)	Wait List T2<br>N = 16<br>Mean (SE)	Wait List T3<br>N = 16<br>Mean (SE)	Effect<br>d (95% CI)	P
**EORTC QLQ-C30**						
Global Health Status	69.6 (3.0)	71.5 (3.0)	75.8 (3.9)	77.9 (3.9)	0.21 (-0.24, 0.65)	.36
Physical Functioning	88.3 (2.7)	89.3 (2.7)	88.7 (3.6)	84.2 (3.6)	-0.26 (-0.70, 0.19)	.25
Role Functioning	84.8 (3.7)	90.5 (3.7)	96.5 (4.8)	91.3 (4.8)	0.02 (-0.42, 0.47)	.92
Emotional Functioning	82.1 (3.6)	83.7 (3.6)	83.6 (4.7)	83.6 (4.7)	0.07 (-0.37, 0.52)	.74
Cognitive Functioning	87.5 (3.4)	84.9 (3.4)	82.6 (4.5)	82.6 (4.5)	-0.12 (-0.57, 0.32)	.58
Social Functioning	82.3 (4.1)	88.1 (4.1)	95.0 (5.3)	90.8 (5.3)	0.07 (-0.38, 0.52)	.75
Fatigue	20.6 (3.6)	24.9 (3.6)	21.2 (4.8)	24.6 (4.8)	0.36 (-0.09, 0.80)	.11
Nausea and Vomiting	1.99 (1.38)	3.91 (1.38)	1.35 (1.79)	0.31 (1.79)	0.06 (-0.38, 0.51)	.78
Pain	20.9 (4.3)	17.7 (4.3)	19.5 (5.6)	22.7 (5.6)	-0.00 (-0.45, 0.44)	.99
Dyspnoea	7.94 (3.05)	7.94 (3.05)	9.30 (3.98)	5.14 (3.98)	-0.17 (-0.61, 0.28)	.46
Insomnia	20.8 (4.9)	18.2 (4.9)	32.0 (6.4)	32.0 (6.4)	-0.07 (-0.52, 0.37)	.74
Appetite Loss	2.8 (2.5)	4.1 (2.5)	9.1 (3.3)	11.2 (3.3)	0.13 (-0.31, 0.58)	.55
Constipation	13.2 (3.6)	17.0 (3.6)	12.0 (4.8)	9.9 (4.8)	0.06 (-0.38, 0.51)	.78
Diarrhoea	9.6 (3.1)	12.2 (3.1)	6.7 (4.1)	6.7 (4.1)	0.10 (-0.35, 0.54)	.67
Financial Difficulties	19.5 (4.4)	9.2 (4.4)	6.8 (5.8)	8.9 (5.8)	-0.31 (-0.76, 0.14)	.17
**CES-D Depression**						
Total Score	7.16 (1.49)	7.12 (1.49)	6.90 (1.98)	6.55 (1.98)	-0.06 (-0.51, 0.38)	.78
**Brief Fatigue Inventory**						
Index	2.03 (0.36)	2.16 (0.36)	1.52 (0.47)	1.79 (0.47)	0.19 (-0.26, 0.63)	.41
**Profile of Mood States**						
Tension	4.32 (0.82)	3.55 (0.82)	3.36 (1.09)	3.59 (1.09)	-0.11 (-0.56, 0.33)	.62
Depression	3.69 (0.99)	3.35 (0.99)	2.99 (1.30)	2.24 (1.30)	-0.17 (-0.62, 0.27)	.44
Anger	3.59 (0.86)	3.47 (0.86)	2.82 (1.13)	3.28 (1.13)	0.06 (-0.39, 0.50)	.81
Fatigue	5.79 (0.78)	4.98 (0.78)	3.83 (1.02)	3.20 (1.02)	-0.27 (-0.71, 0.18)	.24
Confusion	2.36 (0.58)	2.47 (0.58)	2.23 (0.76)	2.23 (0.76)	0.03 (-0.42, 0.47)	.90
Vigour	11.6 (0.8)	12.1 (0.8)	13.3 (1.1)	12.9 (1.1)	0.02 (-0.43, 0.46)	.93
Total Mood Disturbance	8.14 (3.83)	5.73 (3.83)	1.94 (5.04)	1.62 (5.04)	-0.12 (-0.57, 0.32)	.59
**Social Constraints Scale**						
Most Important Person	1.56 (0.13)	1.67 (0.13)	1.59 (0.17)	1.69 (0.17)	0.25 (-0.20, 0.69)	.27
Other People	1.70 (0.14)	1.81 (0.14)	1.66 (0.18)	1.51 (0.18)	-0.05 (-0.49, 0.40)	.83

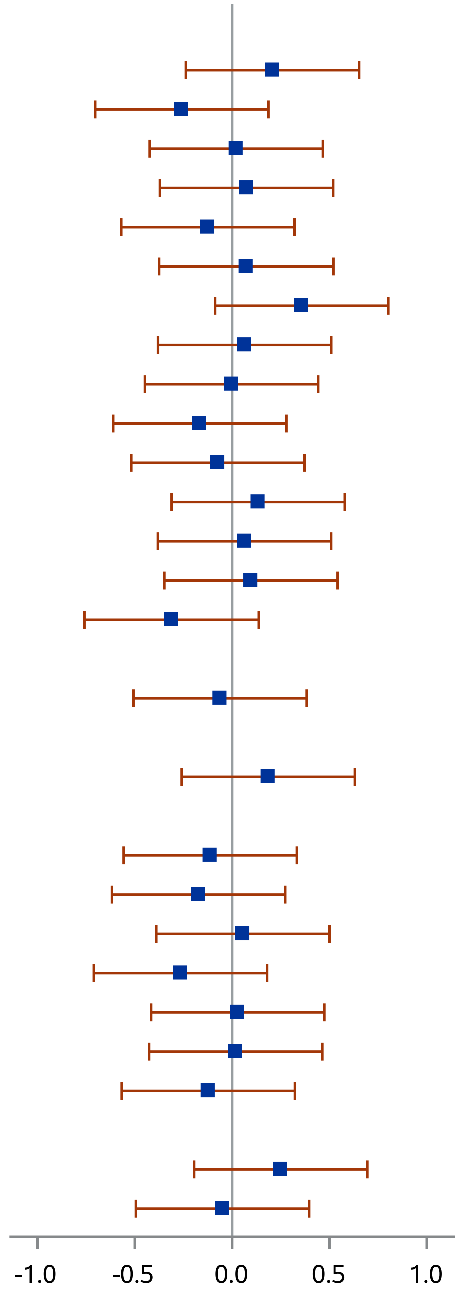

Supplemental Figure 2
